# Supplementary material for: Comparative Characterization of Human Meibomian Glands, Free Sebaceous Glands, and Hair-Associated Sebaceous Glands Based on Biomarkers, Analysis of Secretion Composition, and Gland Morphology
Source: Int J Mol Sci. 2024 Mar 7;25(6):3109. doi: 10.3390/ijms25063109 (PMC10970278; doi:10.3390/ijms25063109)
Supplement: Supplementary file 1 [file ijms-25-03109-s001.zip › Legends supplementary figures.pdf]

Legends supplementary figures

Supplementary Figure S1. Immunohistochemical analysis of human tissue for CK1. The positive antibody reaction was shown by the red coloration. (A)-(C) MG. CK1 is expressed in the superficial layers of the central duct (arrow), but not in the acini (asterisk). (D)-(F) Free SG from nasal wing, (G)- (I) lip, and (J)-(L) EAC. Here, CK1 is expressed in the superficial layer of the central duct (arrow). (M)-(O) Hair-associated SG. CK1 is expressed both in the superficial layer of the central duct (arrow) and in the SG acini (asterisk). No reactivity is visible in the negative control group. Scale bar 100 µm.

Supplementary Figure S2. Immunohistochemical analysis of human tissue for CK8. The positive antibody reaction is shown by the red coloration. (A)-(C) MG. CK8 is expressed in the basal cells of the MG (arrow) and connecting ductules (arrowhead). (D)-(F) Free SG from nasal wing, (G)-(I) lip, (J)-(L) and EAC. CK8 is expressed in the basal cells of the SG (arrow). (M)-(O) Hair-associated SG. CK8 is expressed in the basal cells of the SG (arrow). No reactivity is visible in the negative control group. Scale bar 100 µm.

Supplementary Figure S3. Immunohistochemical analysis of human tissue for CK14. The positive antibody reaction is shown by the red coloration. (A)-(C) MG. CK14 is expressed in the basal layer of the central duct (arrow), basal cells (bc), and differentiating cells (dc) but not in the mature cells (mc). (D)-(F) Free SG from nasal wing, (G)-(I) lip, and (J)-(L) EAC. CK14 is expressed in the basal layer of the central duct (arrow), and bc, dc, and mc. (M)-(O) Hair-associated SG. CK14 is expressed on the basal layers of the duct (arrow), bc, and dc. No reactivity is seen in the mc. Also no reactivity is visible in the negative control group. Scale bar 100 µm.

Supplementary Figure S4. Immunohistochemical analysis of human tissue for N-cadheri n. The positiveantibody reaction is visible by the red coloration. (A)-(C) MG. (D)-(F) Free SG from the nasal wing, (G)-(I) the lip, and (J)-(L) the EAC. N-cadherin is expressed in basal cells of acini (arrowheads) from human eyelid (A)(B), lip (G)(H), and EAC (J)(K). (M)-(O) Hair-associated SG. N-cadherin reactivity occurs in basal cells of acini (arrowheads) and the central duct (arrow). No reactivity was observable in the negative control group. Scale bar 100 µm.

Supplementary Figure S5. Immunohistochemical analysis of human tissue for Desmoglein1. The positiveantibody reaction is visible by the red reaction product. (A)(B)(A')(B') MG. (A)(B) Dsg1 is expressed on the central duct epithelial cells (arrow), basal cells (bc), and differentiating cells (dc), but no staining occurs in mature cells (mc). (A')(B') Dsg1 is expressed in the excretory duct, and equally expressed in bc, dc, and mc. (D)-(F) Free SG from nasal wing, (G)-(I) lip, (J)-(L) EAC.

(M)-(O) Hair-associated SG. Dsg1 is expressed in the excretory duct, and equally expressed in bc, dc, and mc. No reactivity could be seen in the negative control group. Scale bar 100 µm.

Supplementary Figure S6. Immunohistochemical analysis of human tissue for Desmoplakin. The positive antibody reaction shows up as a red reaction product. (A)-(C) MG. (D)-(F) Free SG from nasal wing, (G)-(I) lip, and (J)-(L) EAC. Dsp is expressed in excretory duct epithelial cells (arrow), basal cells (bc), differentiating cells (dc), and mature cells (mc). (M)-(O) Hair-associated SG. Dsp is expressed in excretory duct epithelial cells (arrow), bc, dc and mc. No reactivity is observed in the negative control group. Scale bar 100 µm.

Supplementary Figure S7. Immunohistochemical analysis of human tissue for Desmocollin 3. The positive antibody reaction shows up as a red reaction product. (A)-(C) MG. (D)-(F) Free SG from nasal wing, (G)-(I) lip, and (J)-(L) EAC. Dsc3 is expressed in excretory duct epith elial cells (arrow), basal cells (bc), differentiating cells (dc), and mature cells (mc). (M)-(O) Hair-associated SG. Dsc3 is expressed in excretory duct epithelial cells (arrow), bc, dc and mc. No reactivity could be observed in the negative control group. Scale bar 100 µm.

Supplementary Figure S8. Immunohistochemical analysis of human tissue for Plakoglobin. The positiveantibody reaction shows up as a red reaction product. (A)-(C) MG. (D)-(F) Free SG from nasal wing, (G)-(I) lip, (J)-(L) EAC. Pg is expressed on excretory duct epithelial cells (arrow), basal cells (bc), differentiating cells (dc), and mature cells (mc). (M)-(O) Hair-associated SG. Pg is expressed in excretory duct epithelial cells (arrow), bc, dc and mc. No reactivity can be seen in the negative control group. Scale bar 100 µm.

Supplementary Figure S9. Immunohistochemical analysis of human tissue for E-cadherin. The positiveantibody reaction is visible by the red coloration. (A)-(C) MG. (D)-(F) Free SG from nasal wing, (G)-(I) lip, and (J)-(L) EAC. E-cadherin is expressed in excretory duct epithelial cells (arrow), basal cells (bc), differentiating cells (dc), and mature cells (mc). (M)-(O) Hair-associated SG. E- cadherin is expressed in excretory duct epithelial cells (arrow), bc, dc, and mc. No reactivity is observable in the negative control group. Scale bar 100 µm.

Supplementary Figure S10. Immunohistochemical analysis of human tissue for Claudin5. The positive antibody reaction shows up as a red reaction product. (A)-(C) MG. (D)-(F) Free SG from the nasal wing, (G)-(I) lip and (J)-(L) EAC. (M)-(O) Hair-associated SG from the scalp. Claduin5 does not reveal positive reactivity. In addition, no reactivity is visible in the negative control group. Scale bar 100 µm.

Supplementary Figure S11. Positive control for Claudin 5. Immunohistochemical demonstration of Claudin 5 in human kidney revealing reactivity in sections through nephrons (left) and cells of the glomeruli (right). Scale bar 100 µm.

Supplementary Figure S12. Processing and 3D reconstruction. The figure shows a black and white volumetric image of a woman's upper eyelid reconstructed from 250 sections.
A horizontal cut edge can be seen at the top, a cut edge facing the viewer from which glandular acini of a meibomian gland, stained purple, appear to emerge, and two lateral cut edges. After annotation of the glandular acini, the corresponding structure of interest can be isolated from the histological serial sections. f = female, Mg = Meibomian gland, a = acinus.

Supplementary Figure S13. Evaporator with a dry stream of nitrogen. The evaporator was fabricated by the Machine Shop of the Technical Faculty of FAU (Cauerstraße 5, Erlangen, Germany, contact Mr. Stefan Lange) according to the specifications of Prof. Dr. Igor Butovich (Department of Ophthalmology, the Graduate School of Biomedical University of Texas Southwestern Medical Center, Texas, U.S.A.) and his published work.
