# Supplementary figures and images for: Comparative Characterization of Human Meibomian Glands, Free Sebaceous Glands, and Hair-Associated Sebaceous Glands Based on Biomarkers, Analysis of Secretion Composition, and Gland Morphology
Source: Int J Mol Sci. 2024 Mar 7;25(6):3109. doi: 10.3390/ijms25063109 (PMC10970278; doi:10.3390/ijms25063109)

overview

detail

control group

eyelid

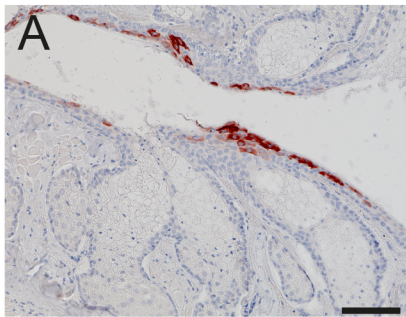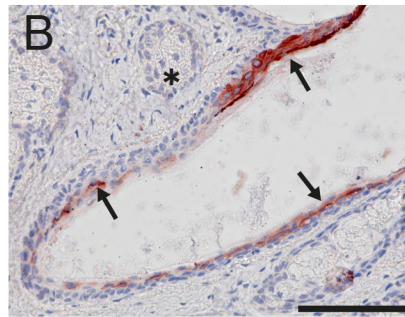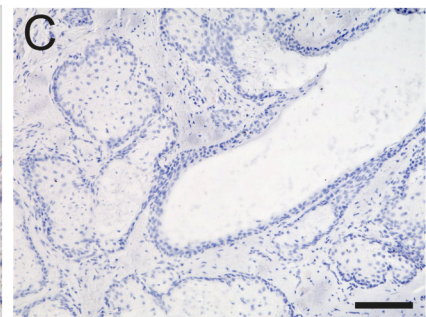

nasal wing

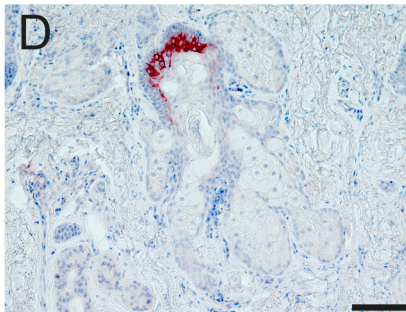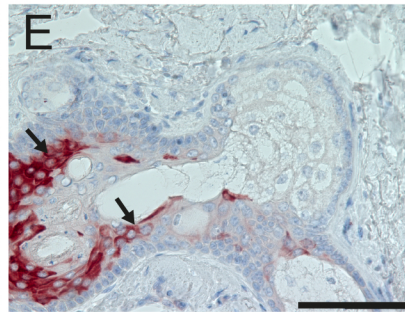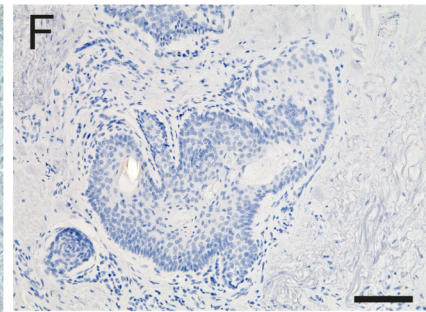

lip

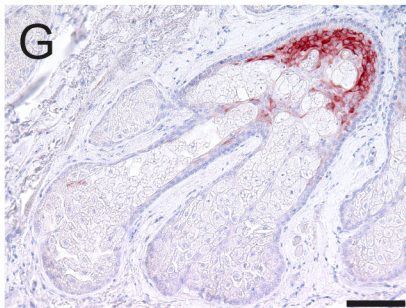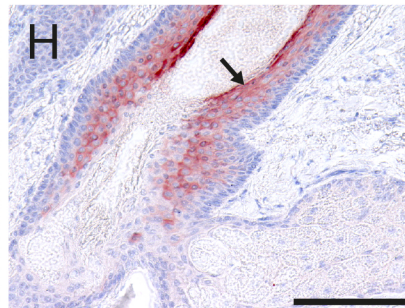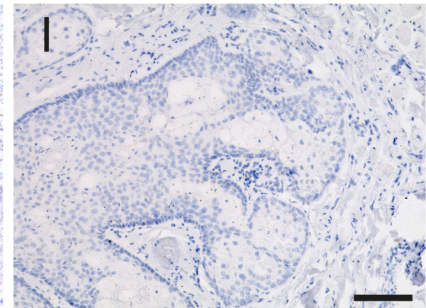

EAC

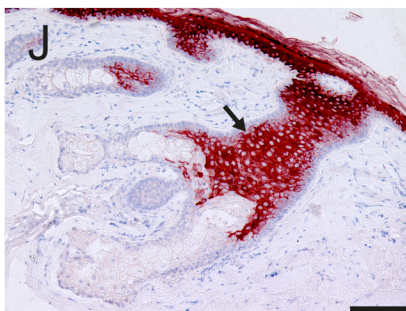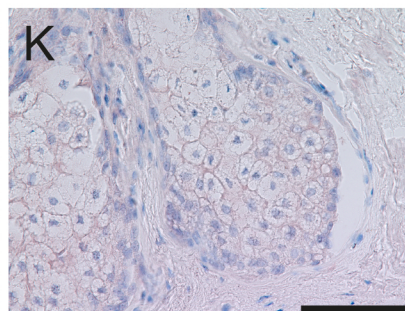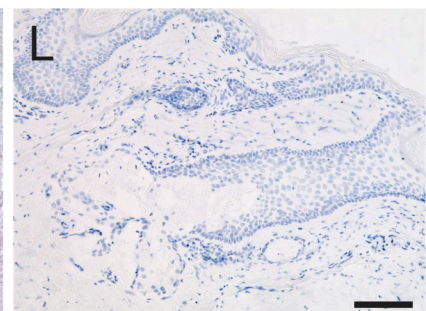

scalp

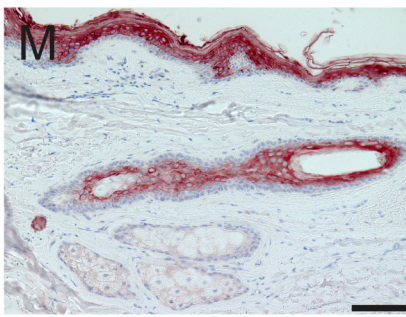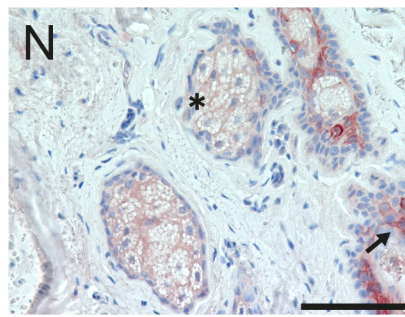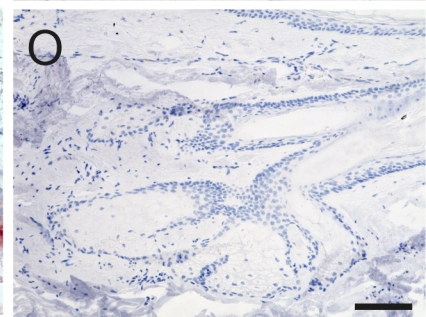

Supplement: Supplementary file 1 [file ijms-25-03109-s001.zip › Supplementary Figure S1 CK1.pdf]

# Claudin5

overview

detail

control group

eyelid

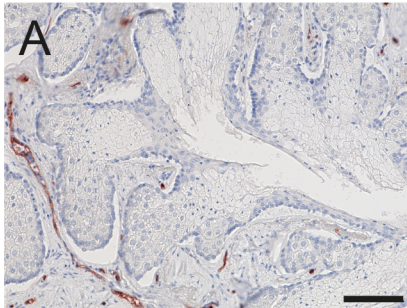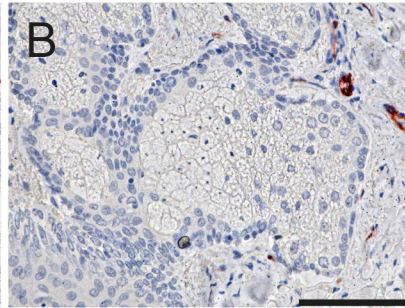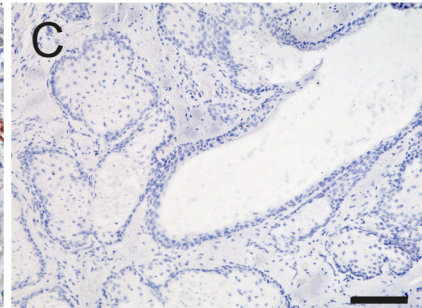

nasal wing

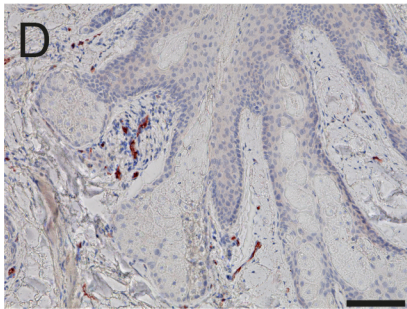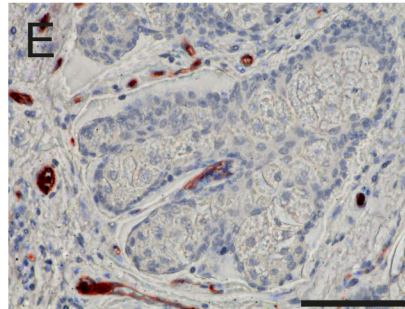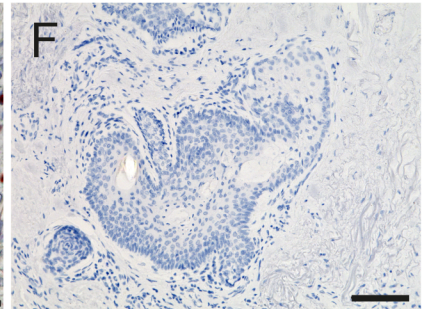

lip

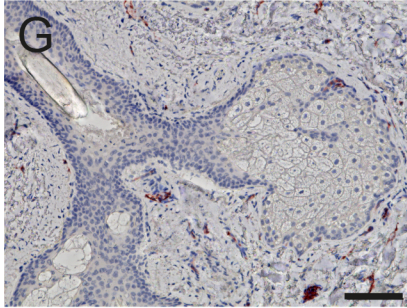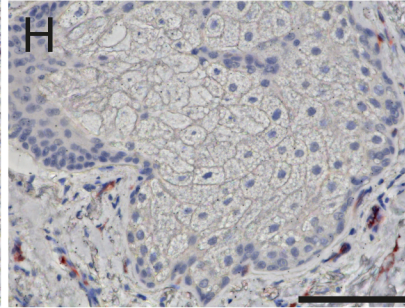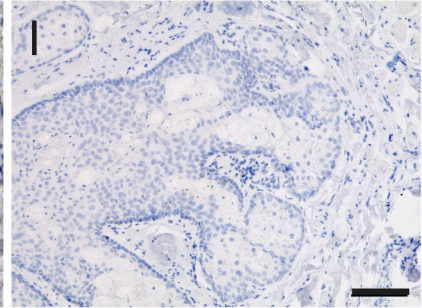

EAC

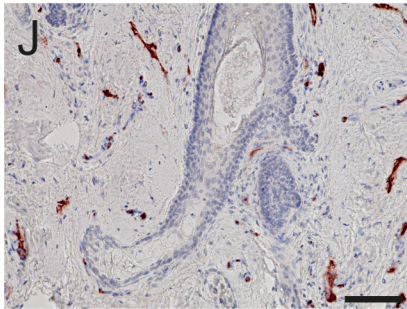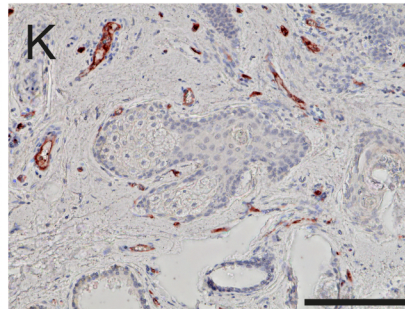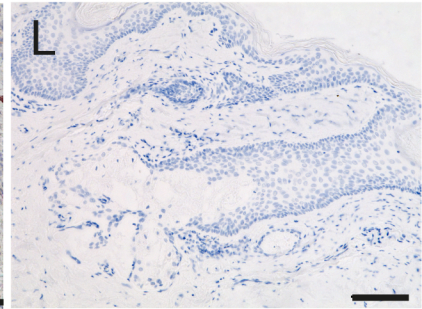

scalp

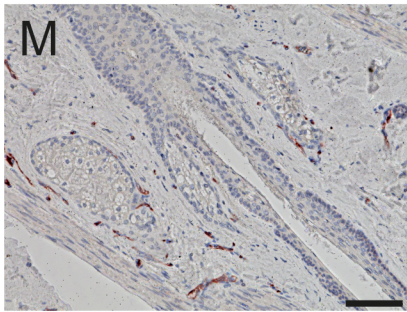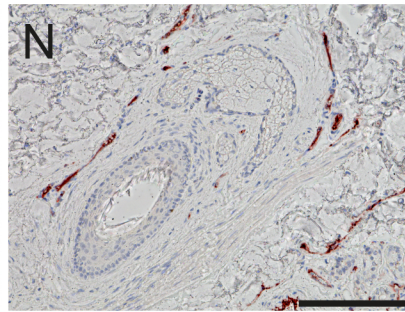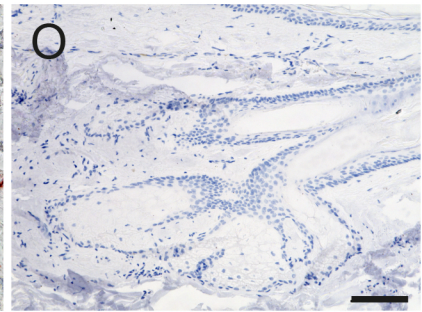

Supplement: Supplementary file 1 [file ijms-25-03109-s001.zip › Supplementary Figure S10 Claudin 5.pdf]

kidney

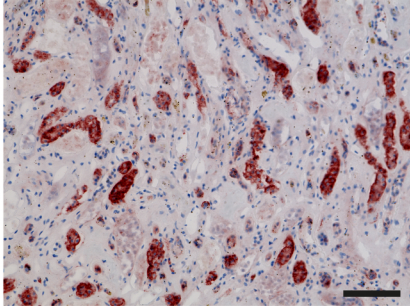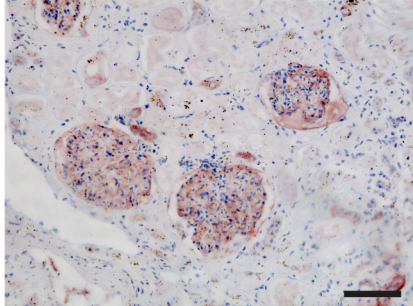

Supplement: Supplementary file 1 [file ijms-25-03109-s001.zip › Supplementary Figure S11 Claudin 5 control kidney.pdf]

# Meibomian gland (f)

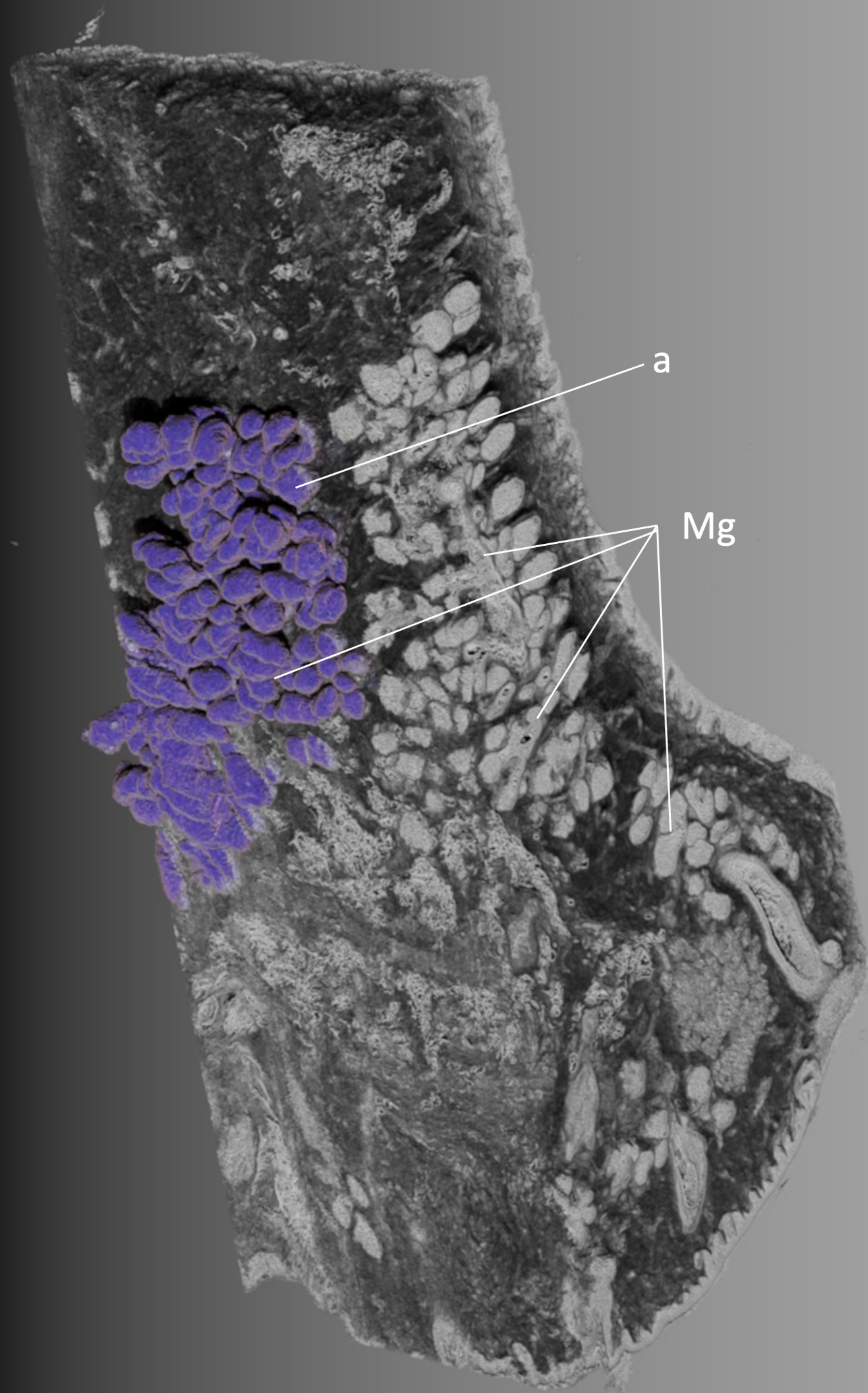

Supplement: Supplementary file 1 [file ijms-25-03109-s001.zip › Supplementary Figure S12 Three dimensional reconstruction.pdf]

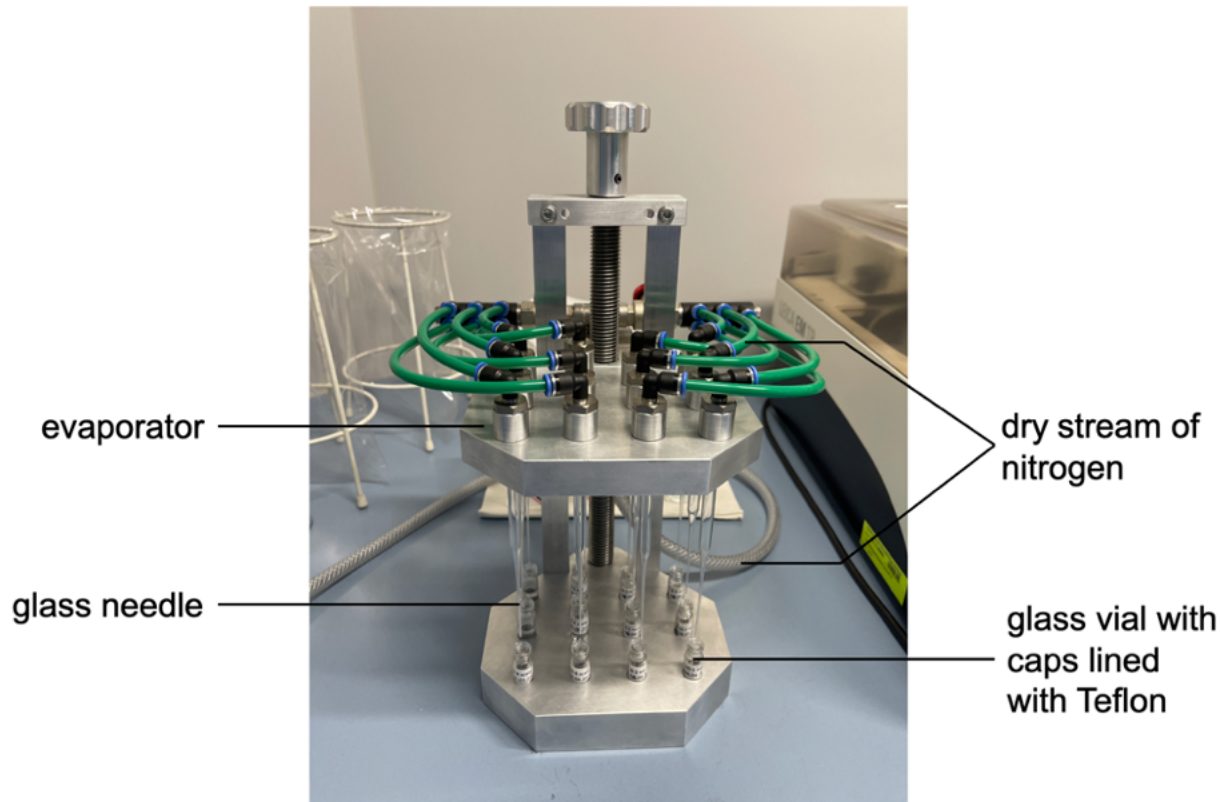

Supplement: Supplementary file 1 [file ijms-25-03109-s001.zip › Supplementary Figure S13 Evaporator.pdf]

# Cytokeratin 8

overview

detail

control group

eyelid

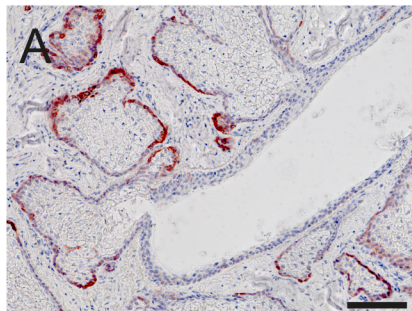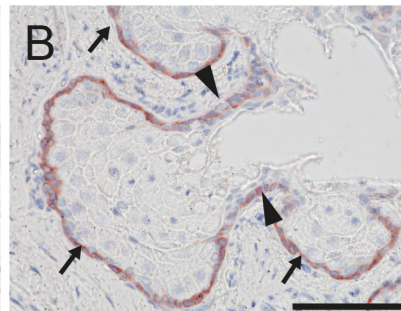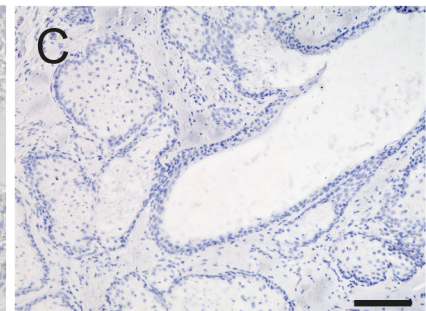

nasal wing

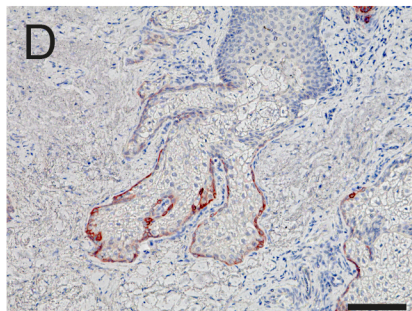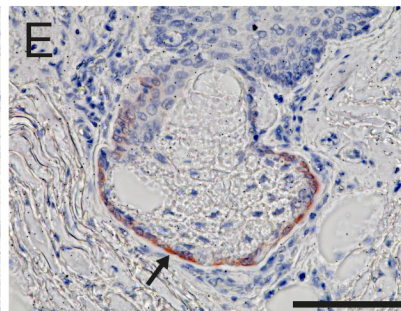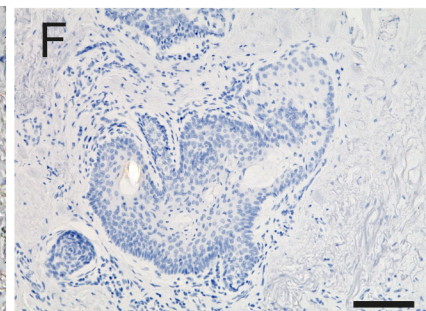

lip

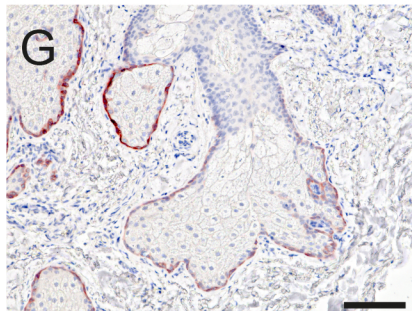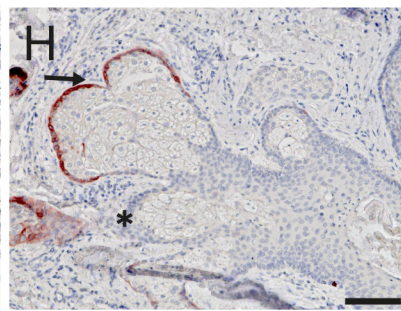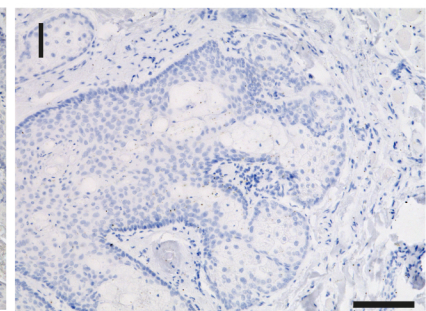

EAC

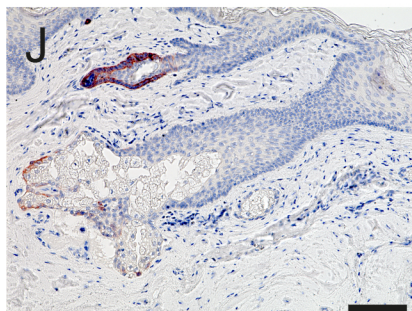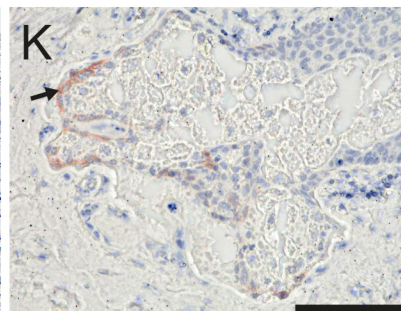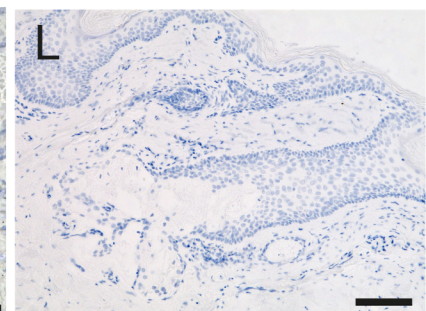

scalp

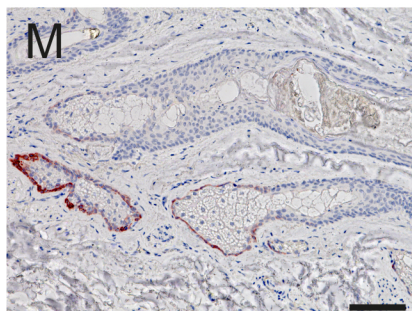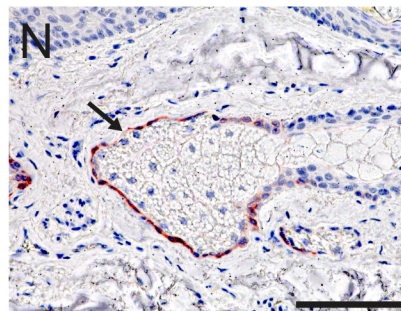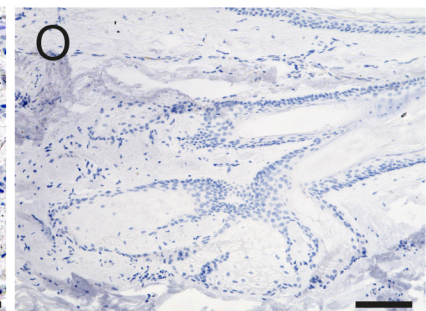

Supplement: Supplementary file 1 [file ijms-25-03109-s001.zip › Supplementary Figure S2 CK8.pdf]

# Cytokeratin 14

overview

detail

control group

eyelid

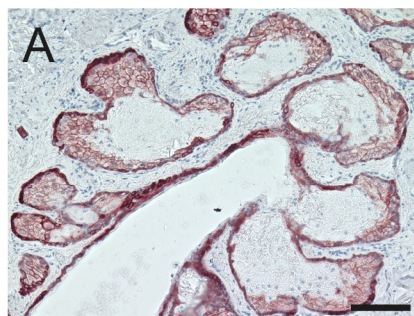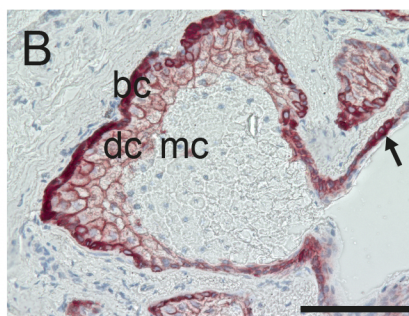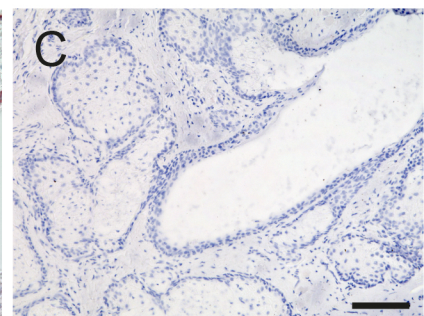

nasal wing

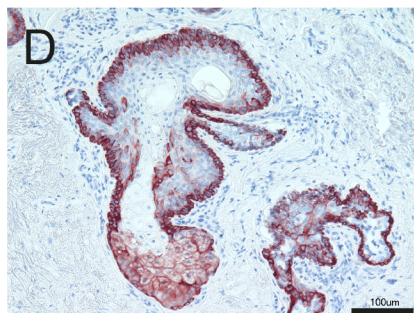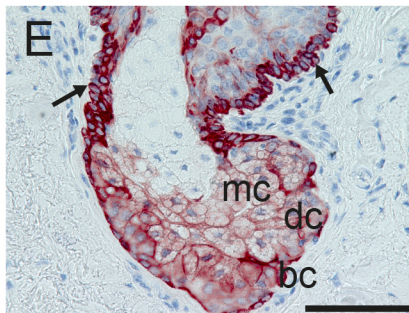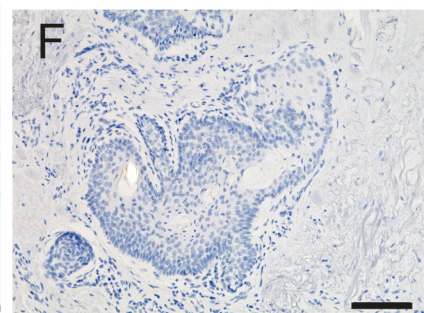

lip

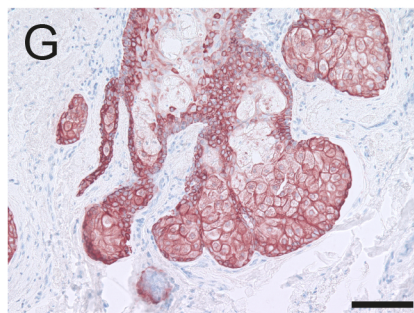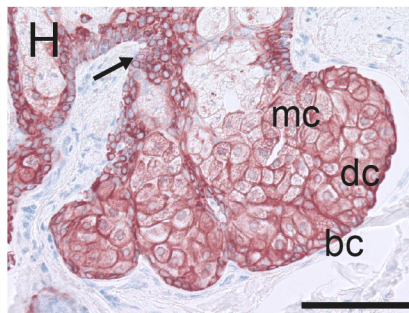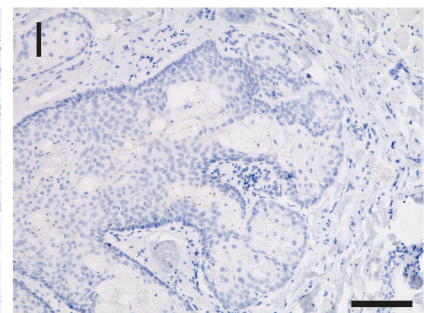

EAC

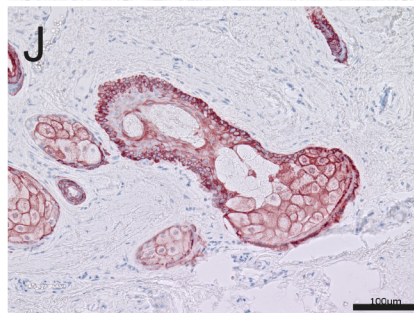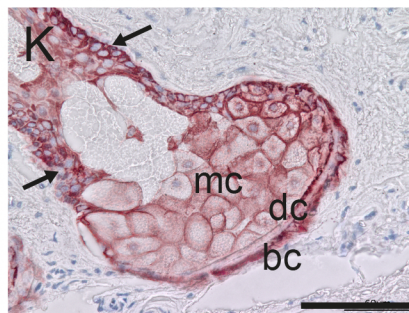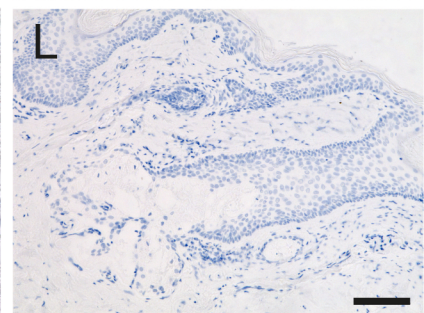

scalp

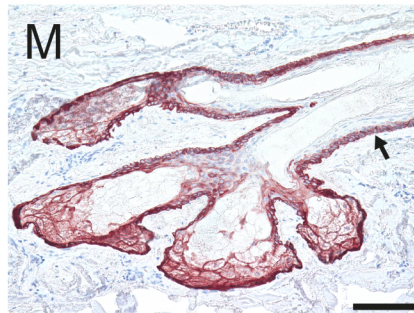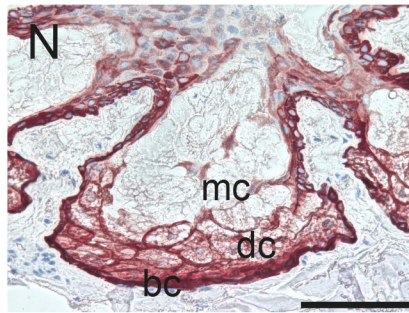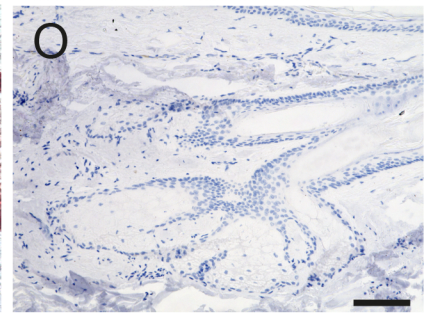

Supplement: Supplementary file 1 [file ijms-25-03109-s001.zip › Supplementary Figure S3 CK14.pdf]

# N-cadherin

overview

detail

control group

eyelid

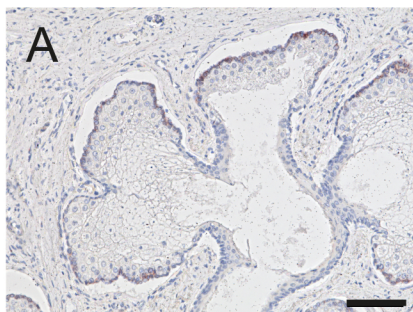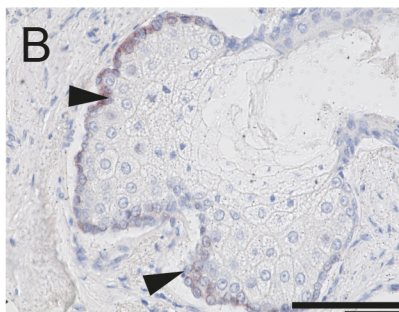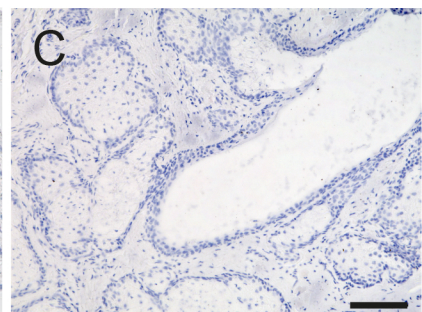

nasal wing

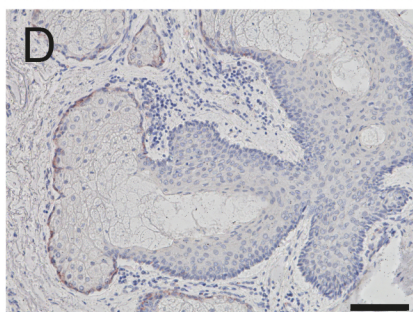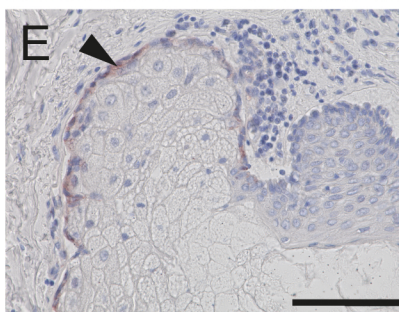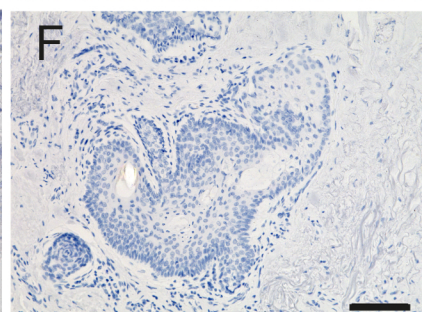

lip

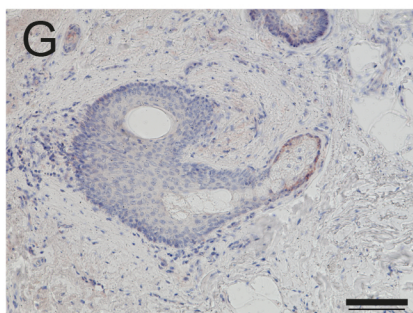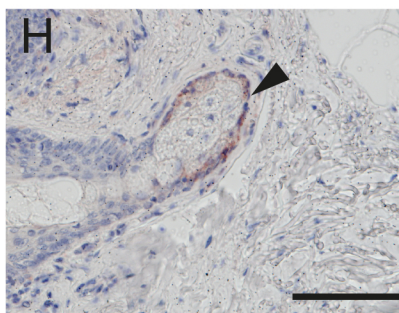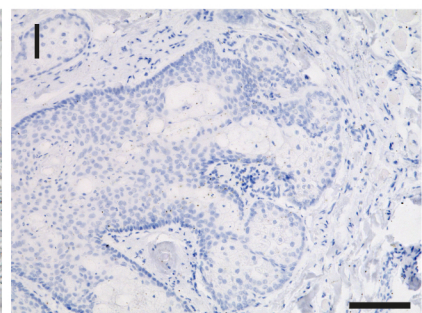

EAC

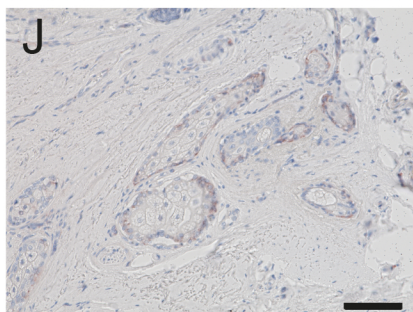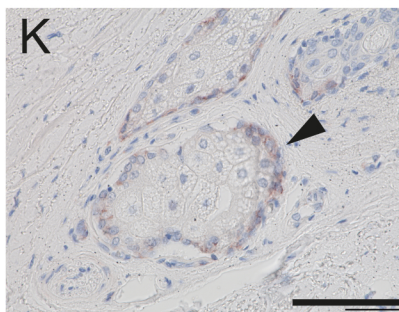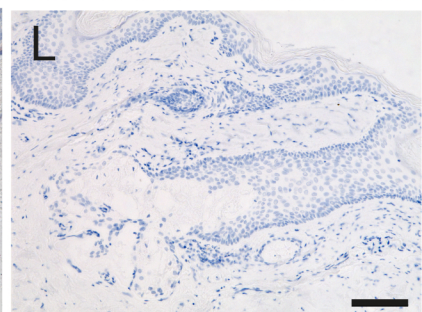

scalp

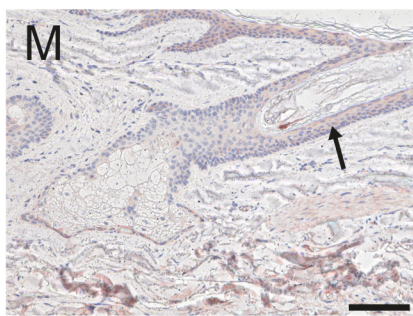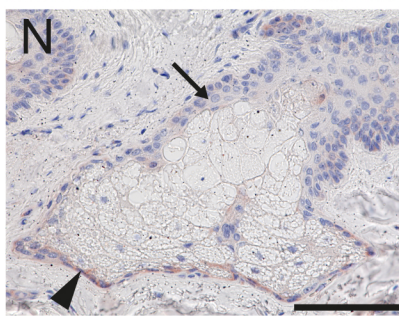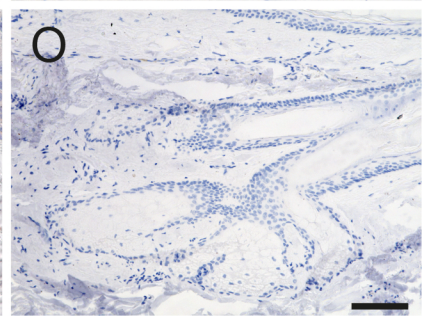

Supplement: Supplementary file 1 [file ijms-25-03109-s001.zip › Supplementary Figure S4 N-cadherin.pdf]

# Desmoglein 1

overview

detail

control group

eyelid

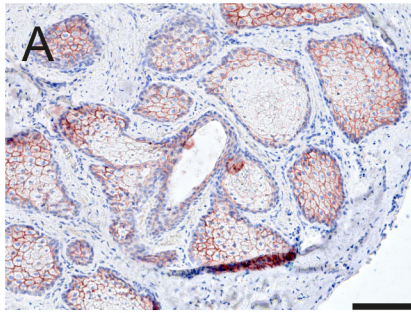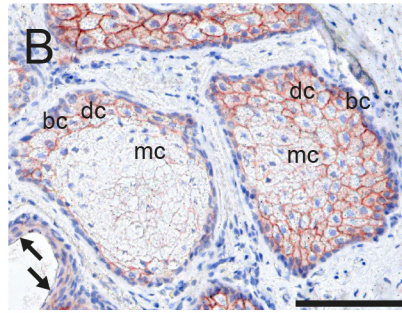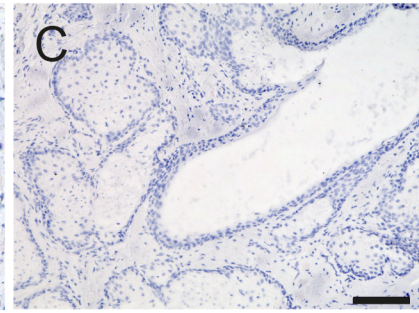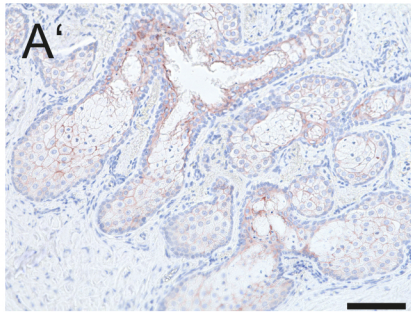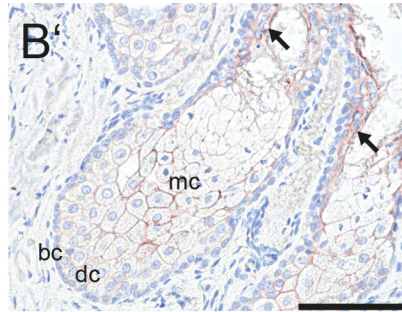

nasal wing

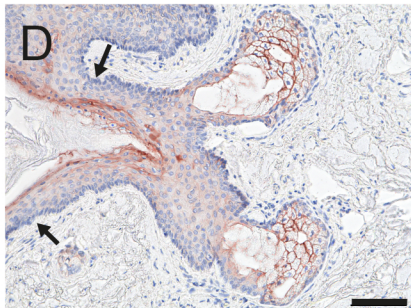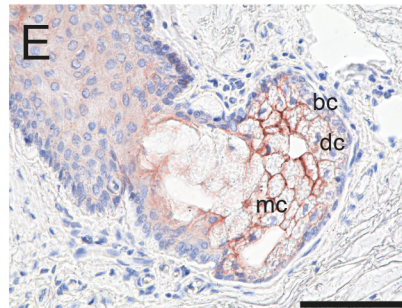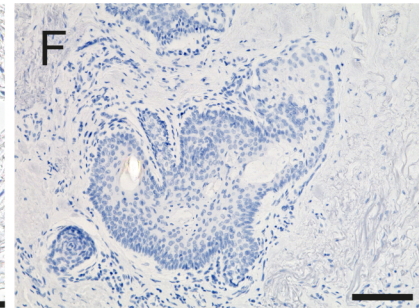

lip

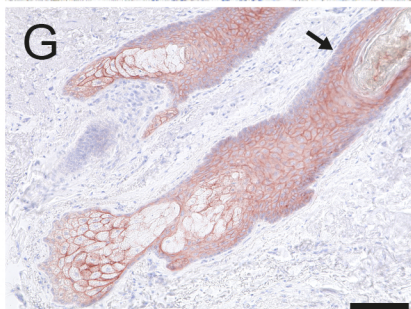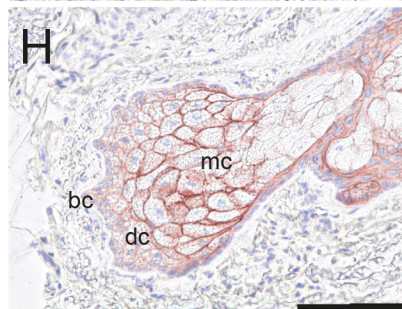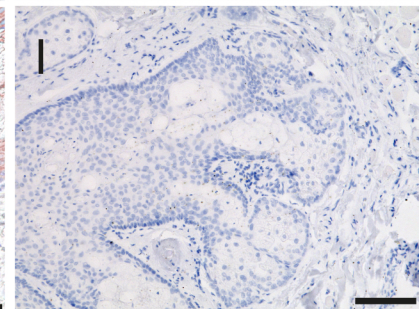

EAC

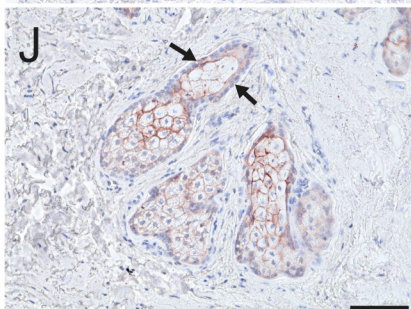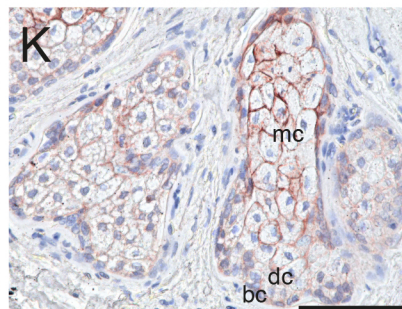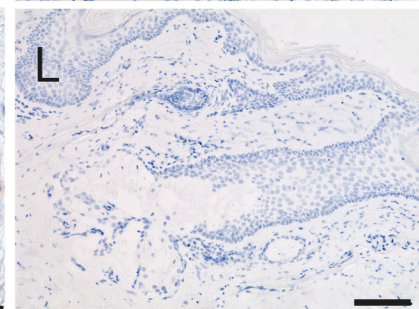

scalp

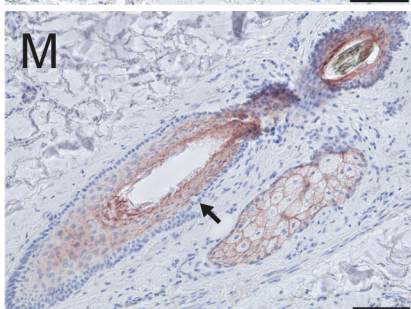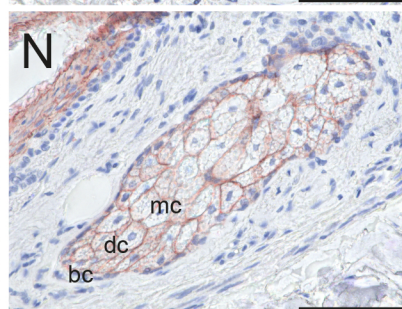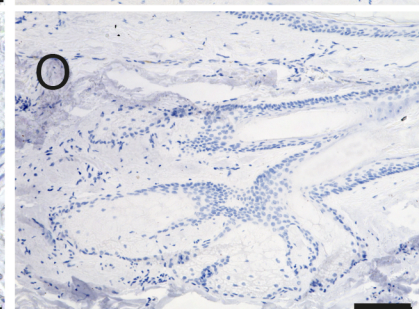

Supplement: Supplementary file 1 [file ijms-25-03109-s001.zip › Supplementary Figure S5 Desmoglein 1.pdf]

# Desmoplakin

overview

detail

control group

eyelid

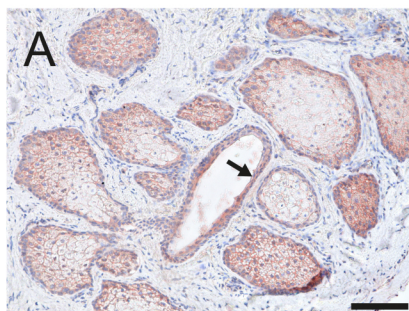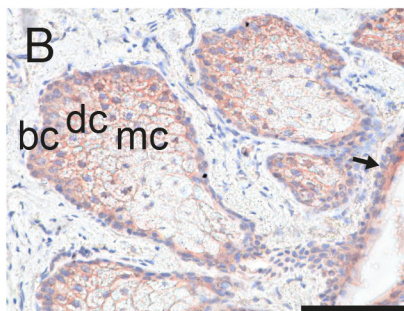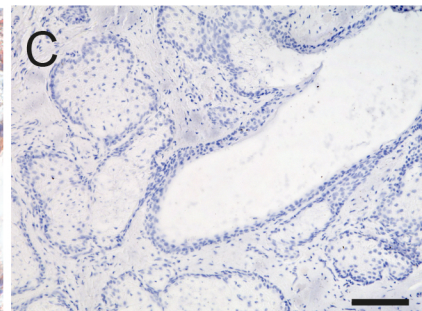

nasal wing

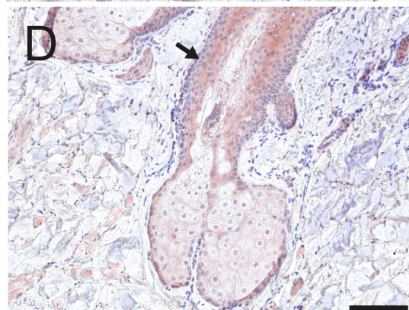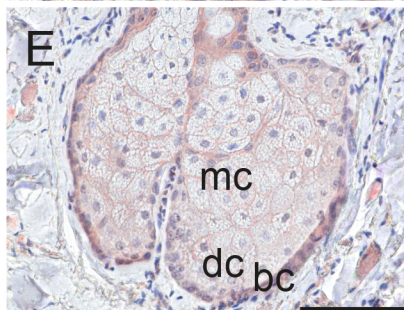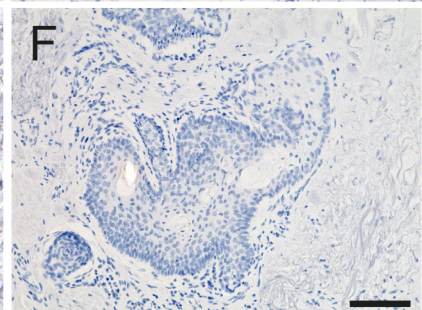

lip

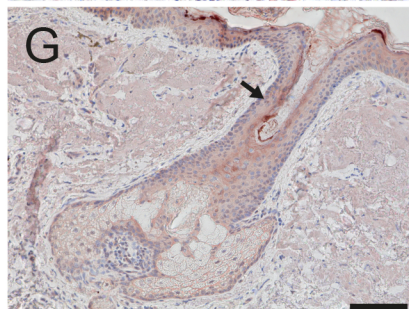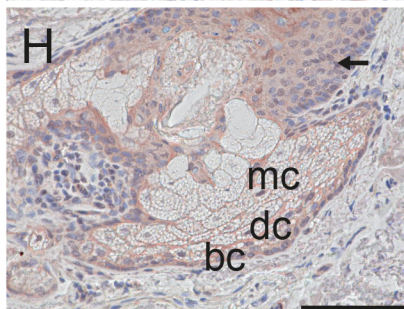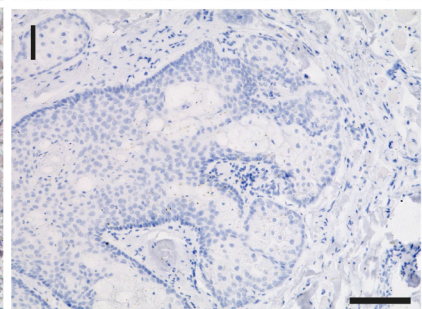

EAC

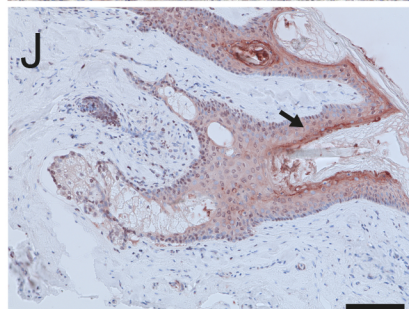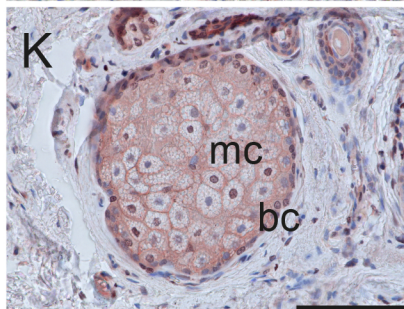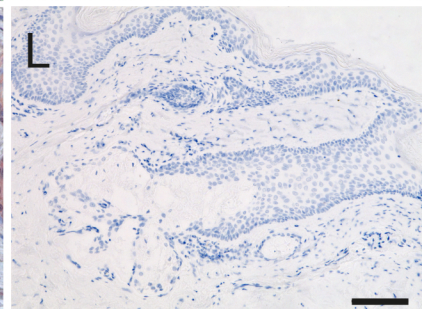

scalp

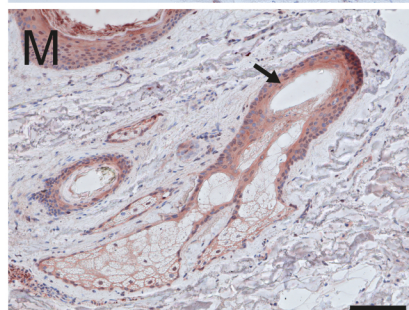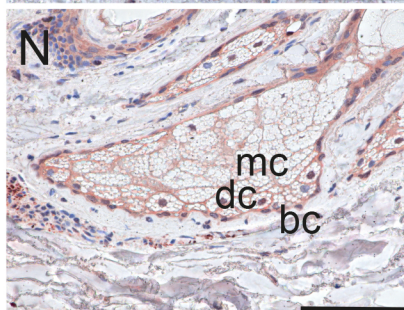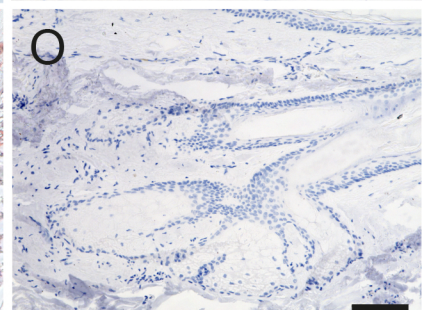

Supplement: Supplementary file 1 [file ijms-25-03109-s001.zip › Supplementary Figure S6 Desmoplakin.pdf]

# Desmocollin 3

overview

detail

control group

eyelid

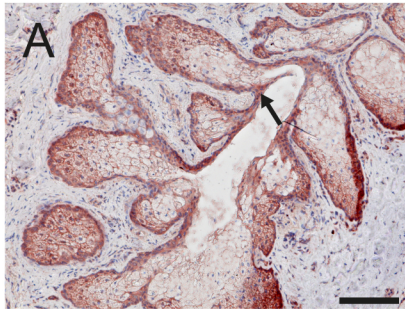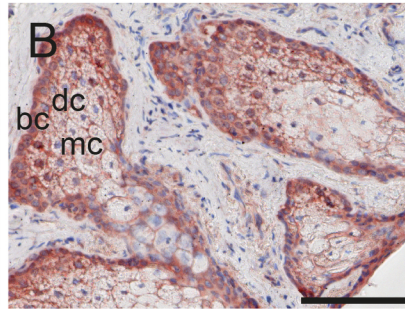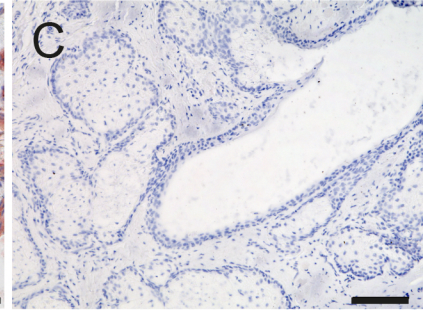

nasal wing

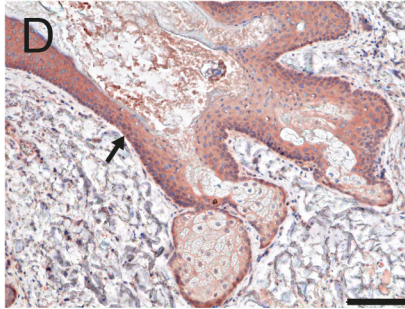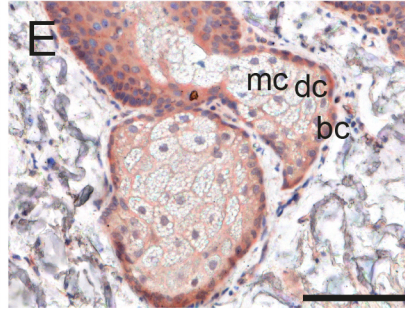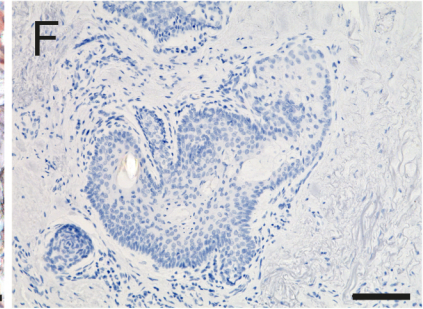

lip

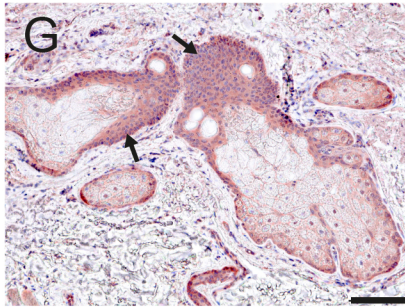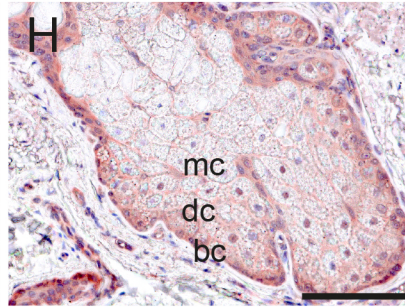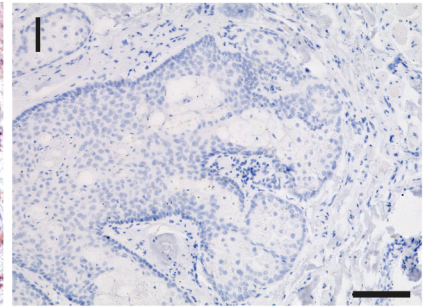

EAC

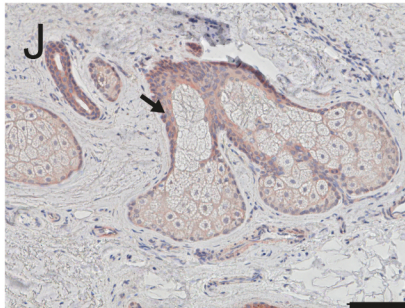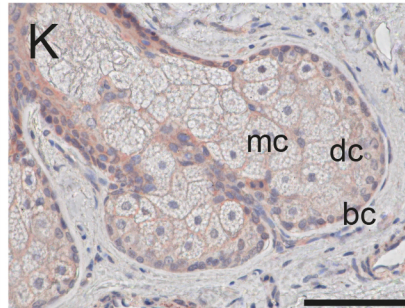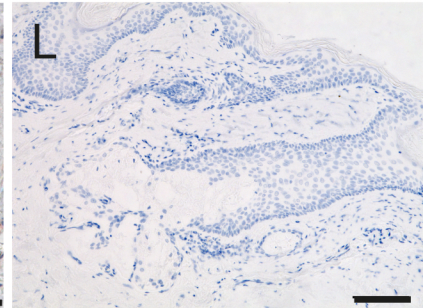

scalp

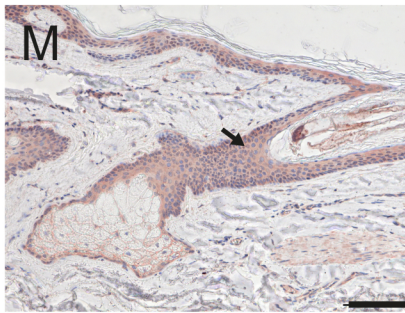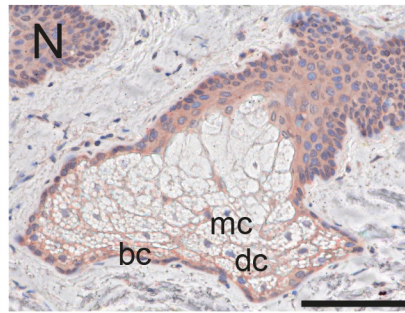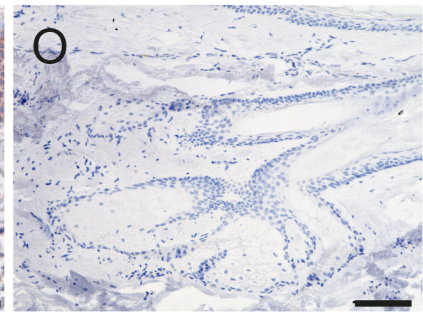

Supplement: Supplementary file 1 [file ijms-25-03109-s001.zip › Supplementary Figure S7 Desmocollin 3.pdf]

# Plakoglobin

overview

detail

control group

eyelid

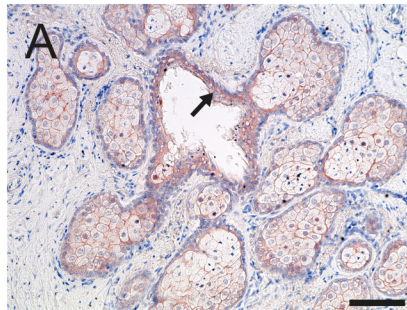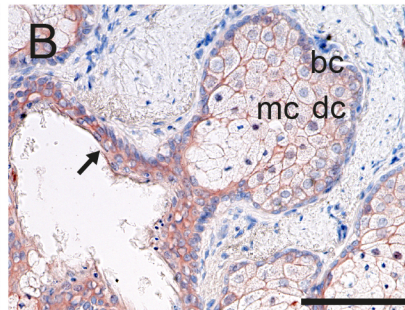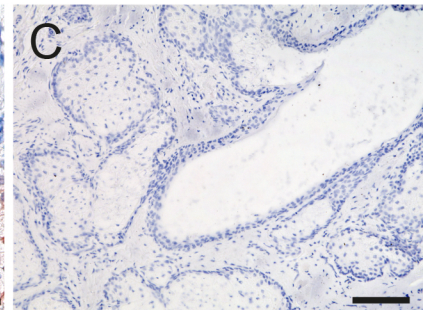

nasal wing

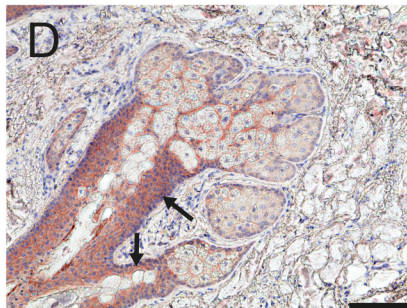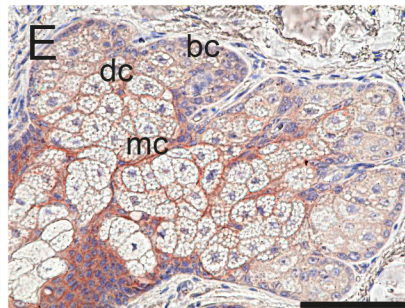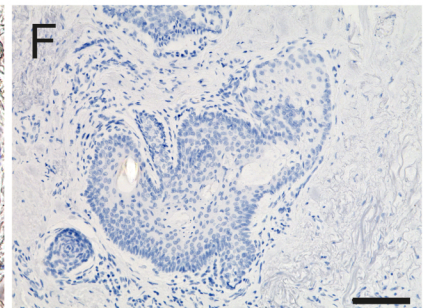

lip

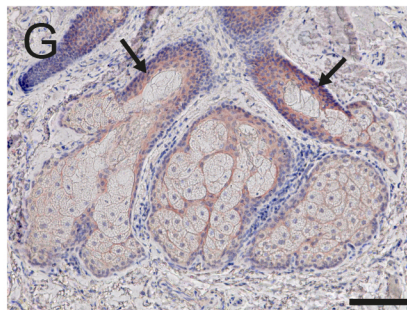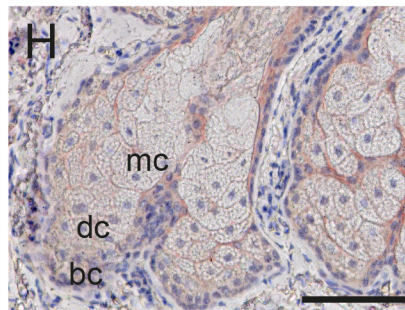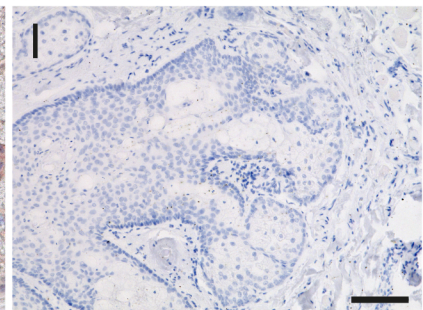

EAC

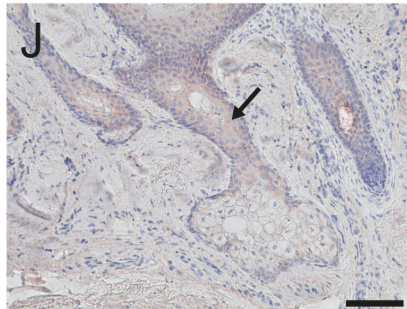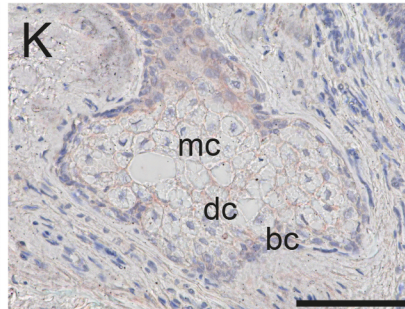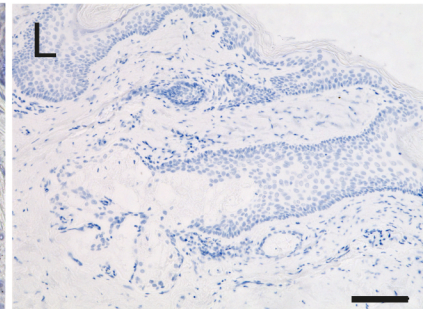

scalp

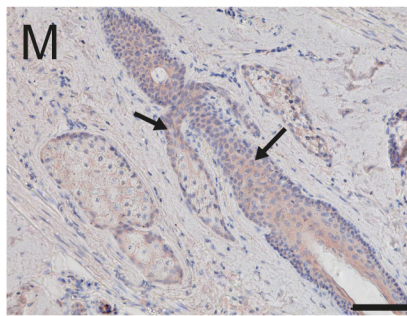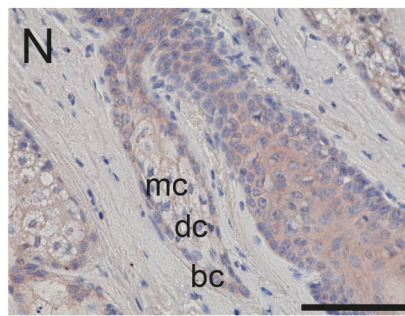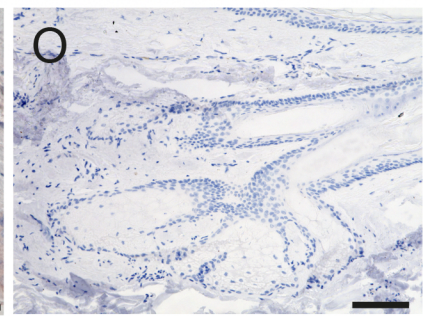

Supplement: Supplementary file 1 [file ijms-25-03109-s001.zip › Supplementary Figure S8 Plakoglobin.pdf]

# E-cadherin

overview

detail

control group

eyelid

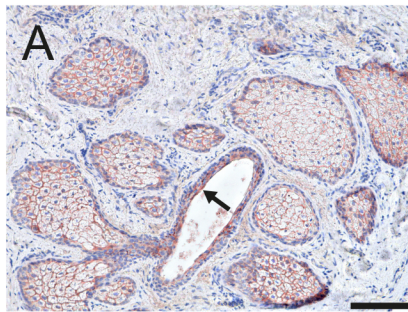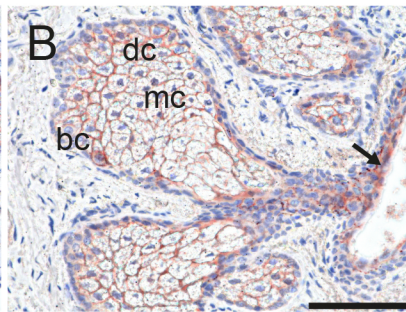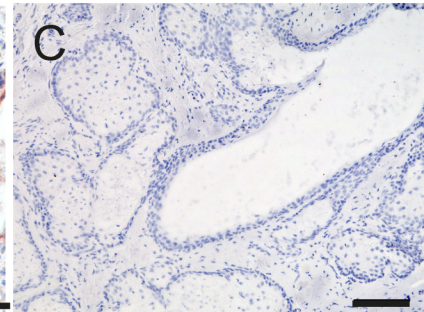

nasal wing

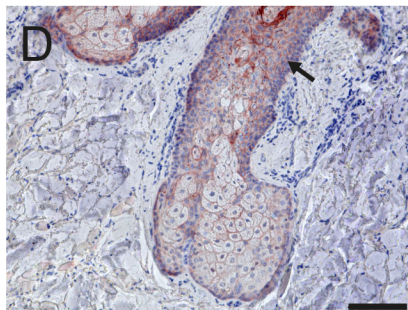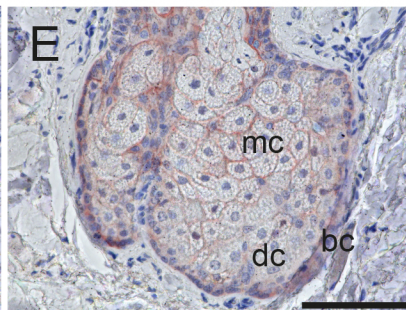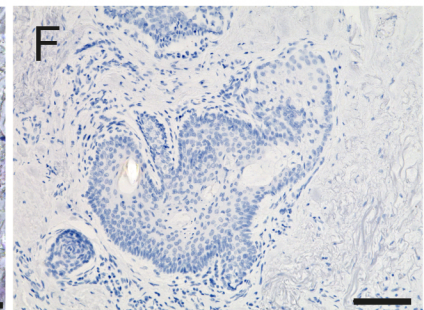

lip

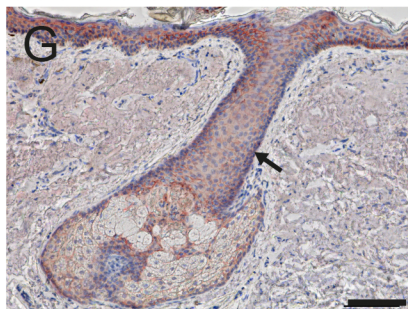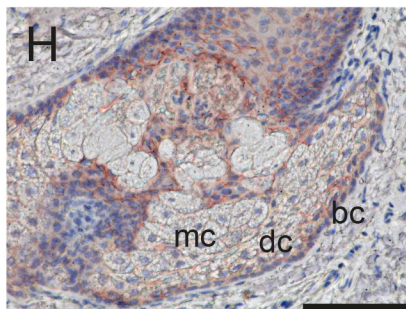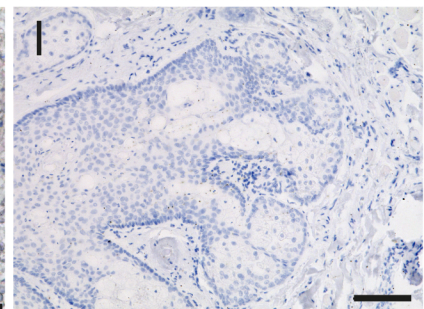

EAC

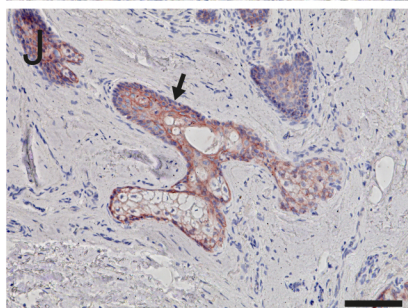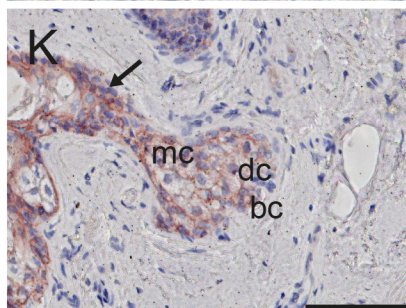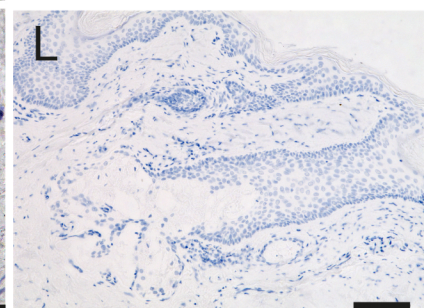

scalp

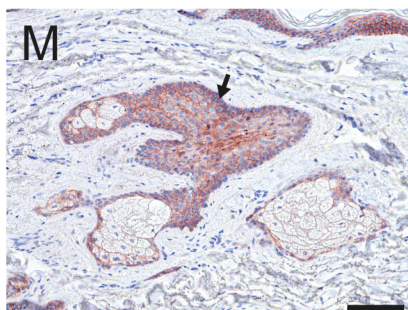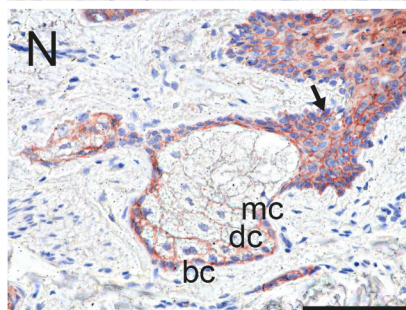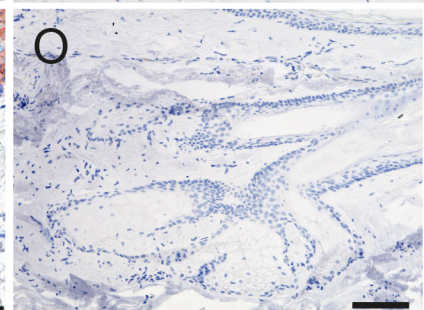

Supplement: Supplementary file 1 [file ijms-25-03109-s001.zip › Supplementary Figure S9 E-Cadherin.pdf]
